# Climate Warming Will Reduce Boreal Forest Litterfall, but the Response Differs Among Plant Functional Types

**DOI:** 10.1002/ece3.73726

**Published:** 2026-05-27

**Authors:** Wai Phyo Thu, Mark Jun M. Alcantara, Gbadamassi G. O. Dossa, Jill Thompson, Douglas Schaefer

**Affiliations:** ^1^ Laboratory of Tropical Forest Ecology Xishuangbanna Tropical Botanical Garden, Chinese Academy of Sciences Mengla China; ^2^ Yunnan Key Laboratory of Forest Ecosystem Stability and Global Change Xishuangbanna Tropical Botanical Garden, Chinese Academy of Sciences Mengla Yunnan China; ^3^ University of Chinese Academy of Sciences Beijing China; ^4^ UK Centre for Ecology & Hydrology Bush Estate Midlothian UK; ^5^ Centre for Mountain Futures Kunming Institute of Botany, Chinese Academy of Sciences Kunming Yunnan China

**Keywords:** carbon cycling, CMIP6, SSP2‐4.5, SSP5‐8.5, stand age, temperature sensitivity

## Abstract

Boreal forests are critical carbon sinks increasingly threatened by climate change. More than two‐thirds of this boreal carbon is stored in soil and litter, highlighting the crucial role of litterfall for carbon and nutrient cycling. Boreal forests have stands of different plant functional types (deciduous, evergreen, and mixed), and we lack information on their contributions to litterfall production. This hinders our understanding of spatiotemporal variations in boreal litterfall production, and thus predictions under future climate scenarios. Here, we synthesized boreal litterfall data from published studies and applied generalized additive models to (1) examine stand age‐ and temperature‐related litterfall dynamics across plant functional types and (2) project future trends under climate change, using two climate scenarios [CMIP6 climate scenarios (SSP2‐4.5 and SSP5‐8.5)]. Mean litterfall production was 1959 kg ha^−1^ year^−1^, with deciduous forests showing the greatest litterfall production. Litterfall production increased with stand age, then declined, peaking at 60 years for evergreen forests and 150 years for mixed forests. Deciduous forests showed no significant age‐related trends in litterfall. Critical temperatures may affect litterfall production; for evergreen forests, the threshold is 5°C, above which litterfall production stabilized, with no threshold for deciduous nor mixed forests. The two CMIP6 climate scenarios predicted widespread declines in boreal litterfall production by the end of the 21st century. The decline in litterfall production was pronounced under SSP5‐8.5, with 70% of boreal forests projected to experience litterfall declines exceeding −20%. Synthesis: Our study highlights the importance of incorporating specific plant functional types and stand age dynamics in predictive models of litterfall production. The findings provide a robust foundation for predicting future changes in boreal litterfall production, thereby advancing the understanding of vegetation‐climate interactions in these forest ecosystems.

## Graphical abstract

We synthesized boreal litterfall data from published studies and applied generalized additive models to examine how stand age‐ and temperature‐related litterfall dynamics varies across plant functional types, and to project future trends under climate change. Litterfall production peaked at different stand ages among plant functional types and showed clear temperature thresholds only in evergreen forests. Future climate projections indicate widespread declines in boreal litterfall production by the end of this century.

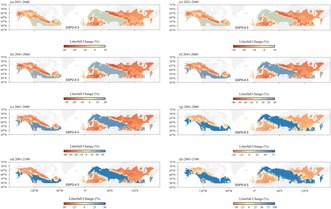

## 1 Introduction

Boreal forest is a large terrestrial biome, accounting for one‐third of the total global terrestrial carbon (C) stock (Pan et al. 2011). This biome is characterized by harsh winters with below‐zero temperature for 6–8 months and short, cool summers (Dial et al. 2022). Climate change is more pronounced in the boreal forests than other forest ecosystems, and they are warming about four times faster than the global average (Rotbarth et al. 2025). The boreal region is projected to experience a temperature rise of 4°C–11°C by the end of this century, coupled with an increase in precipitation (World Bank 2014). Boreal forests support relatively low tree species diversity compared to tropical and temperate biomes (Vankat 2002; Gauthier et al. 2015), and small changes in species diversity can have substantial effects on C sequestration and ecosystem stability (Pearson et al. 2013). Moderate warming and an extended growing season can increase tree species diversity, but extreme warming exceeding 0.65°C per decade negatively affects diversity in boreal forests (Xi et al. 2024). Extreme warming can exceed the thermal tolerance of boreal trees and increase the frequency of forest wildfires, leading to widespread tree mortality (Walker et al. 2019; Wu et al. 2019; Iglesias et al. 2022). Given their relatively low diversity, large C stock, and strong climate sensitivity, boreal forests offer an excellent system to study climate‐ecosystem interaction, without the confounding effects from high biodiversity.

In boreal forests, only 30% of C is in live tissue and dead wood, with more than 70% of the total C stock stored in soil and leaf litter (Scharlemann et al. 2014; Bradshaw and Warkentin 2015). This soil and litter proportion is substantially higher than in temperate (60%) or tropical forests (30%) (Pan et al. 2011), highlighting the crucial role of litterfall (a proxy for C flux) as a primary pathway of C transfer from aboveground biomass (AGB) to soil (Bray and Gorham 1964). Global environmental changes, including elevated CO_2_ (Liu et al. 2005), increased temperature (Raich et al. 2006), and altered precipitation patterns (Zhao and Running 2010), have significantly impacted forest ecosystems including litterfall production and productivity and nutrient dynamics (Kho et al. 2013). This suggests that litterfall fluxes are critical indicators of boreal forest responses to climate change. Understanding litterfall dynamics, therefore, holds critical power for predicting the future trajectory of boreal forest ecosystems and the role of litterfall in global forest C cycling under changing climate conditions.

Previous studies have shown that on a global scale annual litterfall production is primarily regulated by mean annual temperature (MAT) and mean annual precipitation (MAP) (Vogt et al. 1986; Li et al. 2019; Shen et al. 2019). Together, these climatic factors explain about 43% of the variation in global litterfall (Shen et al. 2019). However, annual litterfall responses to climate are likely to differ across plant functional types (PFTs), due to differences in leaf traits and ecological strategies. When compared to evergreen species, deciduous species typically exhibit higher photosynthetic rates per unit leaf area and minimize water loss during drought or low temperature by shedding their leaves (Reich et al. 1997; Baldocchi et al. 2010). Evergreen species have longer leaf lifespans, which are associated with greater leaf construction costs and lower nutrient cycling rates (Chabot and Hicks 1982; Villar and Merino 2001). Notably, evergreen tree species have less ability to acclimate their photosynthetic processes to warming compared to deciduous tree species (Zohner and Renner 2019; Dusenge et al. 2020). These physiological and phenological differences are reflected in PFT‐specific responses to climate, as demonstrated in AGB patterns. For example, globally, higher MAT has a significant positive effect on the initial AGB accumulation in evergreen needleleaf forest, but a negative effect in deciduous broadleaf forest (Chen et al. 2023). Similarly, in the western boreal forest of Canada, net annual AGB change was greater in deciduous broadleaf and early‐successional coniferous forests compared to mixed and late‐successional coniferous forests (Chen and Luo 2015). Previous studies have suggested that litterfall production is strongly linked to AGB because litterfall is largely determined by aboveground stand characteristics (Lohbeck et al. 2015; Chen et al. 2017; Feng et al. 2019; Costa et al. 2020). However, large‐scale studies in boreal regions have generally been based on AGB dynamics (Bradshaw and Warkentin 2015; Liu et al. 2024; Mukhopadhyay et al. 2024; Tompalski et al. 2024; Xu and Hisano 2024) and have often overlooked litterfall production. As a result, there are still gaps in our knowledge about how litterfall production varies across different PFTs, and consequently C cycling will respond to climate warming at the biome scale.

Forest management (plantation vs. natural forests) also plays an important role in shaping litterfall production. Natural forests typically with greater tree species diversity and structural complexity enhanced resource use efficiency and stable ecosystem processes generally have higher productivity and greater litterfall production (Huang et al. 2018; Xu et al. 2021; Gao et al. 2023). In contrast, plantation managed forests often exhibit simplified and more homogenous stand structures, frequently associated with higher tree densities that intensify intraspecific competition and may reduce biomass accumulation (Cheng et al. 2025). These structural and compositional differences can influence litterfall dynamics across forest types. Stand age further modulates litterfall production. Stand biomass accumulation typically peaks in the early stages of development when forests have achieved full canopy closure or maximum leaf area, after which a physiological decline begins as trees grow larger and older (Xu et al. 2012; West 2020). This age‐related decline reflects a shift in resource allocations in older trees, toward defense and maintenance rather than on growth (Herms and Mattson 1992). As a result, older trees generally show slow growth, whereas younger trees (typically under 60 years old) grow much faster (Johnson and Abrams 2009). In addition, the large‐sized trees and the structural complexity which are usually linked with the age increase the maintenance respiration costs and reduce hydraulic efficiency, both of which further constrain growth (Pennisi 2005). Therefore, forest stand age may strongly influence litterfall dynamics as the species community, relative individual tree size, and the number of leaves produced will change with stand age. However, the effect of stand age on litterfall production is uncertain and appears to be region‐ and species‐specific. For instance, annual litterfall production in boreal pine forests in Finland displays no clear trend with stand age (Starr et al. 2005), whereas in boreal forests of Ontario, Canada, the trend in litterfall production increases with stand age, peaking at 98 years (Chen et al. 2017). The relationship between litterfall and stand age may also differ by species composition and PFTs. For instance, before 35 years old, broadleaf evergreen forest exhibits higher AGB accumulation rates than broadleaf deciduous forest, but after 35 years this trend reverses (Chen et al. 2023). Although forest stand age‐related trends in AGB have been well‐studied and synthesized regionally and globally (Cook‐Patton et al. 2020), how stand age affects litterfall production across PFTs in boreal ecosystems remains a key knowledge gap. This gap constrains our understanding of age‐dependent litterfall patterns and their role in boreal C cycling.

Previous studies have shown that moderate climate warming (1°C–2°C) can enhance boreal tree growth by extending the growing season, whereas stronger climate warming (3°C–4°C) may offset these gains and lead to growth declines due to increased physiological stress and disturbance risk (D'Orangeville et al. 2018). The Shared Socioeconomic Pathways (SSPs) provide a framework that integrates greenhouse gas emission trajectories, which increase climate warming, with socioeconomic development, to assess potential responses to future climate change (Gurney et al. 2022). Among these SSPs, SSP2‐4.5 represents an intermediate scenario, with global temperatures projected to increase by approximately 2.7°C by the end of the century, whereas SSP5‐8.5 represents a high‐emission scenario associated with substantially greater warming of around 4.4°C (Masson‐Delmotte et al. 2021). These scenarios are particularly relevant for boreal forests, where ecosystem processes are strongly temperature‐limited and highly sensitive to climate change. Given that litterfall is closely linked to primary productivity and C cycling, examining its response under these scenarios provides a meaningful way to assess potential responses of boreal ecosystems to future climate change.

In this study, we aimed to investigate annual litterfall production across PFTs in boreal forests and the spatiotemporal dynamics of litterfall production, in order to understand their impact on C dynamics under future climate scenarios (SSP2‐4.5 and SSP5‐8.5). Specifically, we set the following objectives for our investigation into boreal forests: (a) Quantify differences in annual litterfall production among plant functional types (PFTs; deciduous, evergreen, and mixed forests) and between forest management types (plantation vs. natural forests) in boreal ecosystems; (b) Evaluate how litterfall production responds to stand age and climatic variables across plant functional types and; (c) Predict spatiotemporal changes of litterfall production under different climate warming scenarios. We tested the following hypotheses: (i) Boreal deciduous forests will produce significantly higher annual litterfall, because of their higher specific leaf area, shorter leaf lifespan, and greater leaf turnover than boreal evergreen forests. (ii) Litterfall production will follow a unimodal relationship with stand age across plant functional types, peaking during the intermediate stages before declining in litterfall in older forests due to increased maintenance respiration costs and resource‐based physiological constraints limit growth. (iii) Deciduous, evergreen, and mixed forests will exhibit different litterfall production responses to mean annual temperature because of their different temperature sensitivities and the duration of their growing seasons. (iv) Under SSP2‐4.5, boreal forest litterfall production is expected to increase as a result of an extended growing seasons, whereas under SSP5‐8.5, it may decline as climate warming exceeds the thermal tolerance of boreal tree species.

To investigate our hypotheses, we first carried out a literature review and synthesis of published data on annual litterfall production across boreal forest PFTs. We then conducted a unique and comprehensive analysis of these data to explore how climate, stand age, and PFTs interact to shape litterfall dynamics. Unraveling these complex interactions will be critical for predicting future changes in litterfall production and their feedback to the global C cycle.

## 2 Materials and Methods

### 2.1 Data Compilation

To compile published data for our analysis, we conducted a preliminary search for peer‐reviewed publications in the ISI Web of Science database (http://isiknowledge.com) on June 1, 2025. We then used the *litsearchr* R package to refine the search keywords (Grames et al. 2019). The following search terms were used:

(“leaf* fall*” OR “aboveground* litter*” OR “annual* litter*” OR “biomass* litter*” OR “broadleaf* litter*” OR “canopi* litter*” OR “conifer* litter*” OR “foliar* litter*” OR “forest* litter*” OR “fresh* litter*” OR “litter* accumul*” OR “litter* addit*” OR “litter* biomass*” OR “litter* C*” OR “litter* compon*” OR “litter* composit*” OR “litter* deposit*” OR “litter* dynam*” OR “litter* fraction*” OR “litter* input*” OR “litter* product*” OR “litter* quantiti*” OR “litterfal* biomass*” OR “litterfal* dynam*” OR “litterfal* input*” OR “litterfal* nutrient*” OR “litterfal* product*” OR “litterfal* rate*” OR “needl* litter*” OR “plant* litter*”) AND (“boreal* forest*” OR “taiga forest*”).

The search produced 384 publications. To minimize publication bias, only studies that met the following criteria were included in the dataset: (1) litterfall data were collected directly using litter traps; (2) total annual litterfall production was reported; (3) the study was carried out in regions between 50° and 70°N latitude (Pfadenhauer and Klötzli 2020). After applying the selection criteria, we reviewed 57 publications.

**FIGURE 1:**
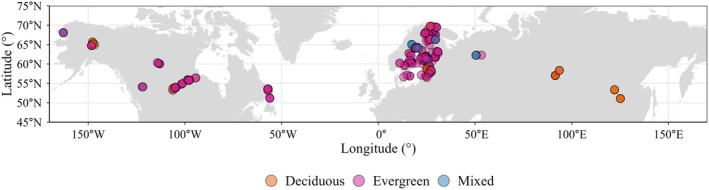
Map of litterfall sampling sites across the boreal forests. Each point (*n* = 277) represents a study location compiled from the literature and is colored according to plant functional types (orange = deciduous forests, pink = evergreen forests, blue = mixed forests).

From the 57 publications we prepared a dataset that included systematically compiled annual litterfall production (kg ha^−1^ year^−1^), including its components (leaves/needles, twigs (< 2.5 cm in diameter), bark, and reproductive parts). We also manually extracted the following: (1) climatic and geographic variables: MAT (°C), MAP (mm year^−1^), latitude, longitude, elevation (m), slope gradient, and country; (2) forest stand characteristics: forest management types (plantation vs. natural), three dominant species, stand age (years), stand density (number of trees per hectare), basal area (m^2^ ha^−1^), mean diameter at breast height (DBH, cm), and mean canopy height (m) for the entire forest stands; (3) study design: study period, number of traps, collection frequency, trap size, mesh size, and sampling design (systematic vs. random). These data were collected directly from the main text, tables, or Supporting Information of each publication. We used WebPlotDigitizer 5 to extract values when data were only presented graphically in figures. For studies reporting multiple locations or experimental treatments, each unique site or treatment was recorded as an independent observation to maintain the spatial resolution in our analysis. Missing geographic coordinates were obtained using Google Earth Pro. Missing MAT and MAP were derived from WorldClim 2.1 at 1 km resolution based on the geographic location of each sampling site (Fick and Hijmans 2017). Forest stand age data were extracted directly from site descriptions and associated metadata of the original publications. Of the 57 articles reviewed, 43 (75.4% of the data) reported stand age data. After compiling the data, litterfall values exceeding two standard deviations from the overall mean were considered outliers and those data points (*n* = 3) were removed to reduce the error during data collation. After outlier removal, the final dataset comprised 277 observations, including 205 evergreen stands, 49 deciduous stands, and 23 mixed stands (Figure 1).

PFTs in our analysis were classified based on the relative dominance of the three dominant species and forest type information provided in each study. Specifically, stands where evergreen species accounted for more than 75% dominance were categorized as evergreen forests, deciduous forests if deciduous species exceeded 75% dominance, and mixed forests for stands with dominance between 25% and 75% for both types (Dataset S1). To ensure taxonomic consistency, we verified and standardized nomenclature of all species using the World Flora Online database.

### 2.2 Litterfall Production Under Historical Climate Conditions and Future Climate Projections

To predict litterfall production under future climate scenarios, we employed two global climate models (GCMs): EC‐Earth3‐Veg and MPI‐ESM1‐2‐HR from the Coupled Model Intercomparison Project Phase 6 (CMIP6) (Eyring et al. 2016; O'Neill et al. 2016). We averaged the climate data (MAT and MAP) from these two models to reduce the potential variability and random errors relying on a single GCM. These models were chosen for their complementary strengths: EC‐Earth3‐Veg incorporates dynamic vegetation modeling and climate‐vegetation feedback mechanisms (Döscher et al. 2022), while MPI‐ESM1‐2‐HR provides high‐resolution climate data with an accurate representation of regional climate patterns (Müller et al. 2018).

We focused on two climate scenarios from the CMIP6 framework: SSP2‐4.5 and SSP5‐8.5, representing intermediate and high‐emission scenarios, respectively. Climate data (MAT and MAP) for these scenarios, along with the historical baseline (1970–2000), were obtained from WorldClim 2.1, which provides downscaled and bias‐corrected projections for ecological applications (Fick and Hijmans 2017). The 1970–2000 period was chosen as the historical climate baseline because it is the standard historical reference period for these types of climate projections, ensuring direct comparability for assessing future climate impacts (Fick and Hijmans 2017). Moreover, 47% of our compiled dataset (1974–2024) falls within this baseline period (1970–2000; Figure S1).

It is important to note that we focused exclusively on how future changes in temperature and precipitation will alter the patterns of annual litterfall production, while keeping other predictor variables such as PFTs, forest management types, and stand age constant. We also did not account for any potential changes in vegetation and soil variables under the climate change scenarios.

### 2.3 Land Cover Dataset

The land cover data was derived from Moderate Resolution Imaging Spectroradiometer‐MODIS land cover (MCD12Q1.061) at 500 m resolution (https://zenodo.org/records/8367523). We reclassified the original land cover types into a binary classification of forests and non‐forests (Table S1).

### 2.4 Statistical Analysis

We applied generalized additive model (GAM; Hastie and Tibshirani 1990) to model nonlinear responses of litterfall production to the set of predictor variables including climatic factors, forest management types, elevation, latitude, PFTs, and stand age. GAM was fitted using *gam* function from the “mgcv” package with a Gamma distribution and a log link function to accommodate the positive, continuous nature of litterfall data (Wood 2017). We trained models to predict litterfall production incorporating a full set of predictors. Country was included as a random effect to account for potential nonindependence among observations within countries. Due to 50 missing values for stand age, the global model was trained on a subset of 227 data points. To minimize the effects of high multicollinearity among variables on model performance, the variance inflation factor (VIF) was calculated for all predictor variables using *vif* function from the “car” package, and all predictors had VIFs below a threshold of 5 (Table S2). The best models were selected based on the Akaike information criterion (AIC; ∆AIC < 2) by using the *dredge* function from “MuMIn” package (Bartoń 2023). Finally, we selected the best 7 models (Table S3). Model performance was validated through residual checks (Figure S2) using *appraise* function from the “gratia” package (Simpson 2019) and *k.check* function from the “mgcv” package for every model (Table S4). The best performing model was selected based on its higher explanatory power and the inclusion of key ecological and spatial drivers (Table S5). The significance of the smooth terms was assessed using *anova.gam* function from the “mgcv” package. To evaluate differences in litterfall production among PFTs and forest management types, post hoc pairwise comparisons were conducted using Estimated Marginal Means (EMM) and were performed with the *emmeans* function of the “emmeans” package with Tukey's adjustment for multiple testing (Lenth 2024).

In addition to the full predictor models, we developed a climate‐focused GAM to isolate the direct effects of MAT and MAP on litterfall production (Figure S3). This model was calibrated using observed climate and litterfall data from the full dataset (*n* = 277). We subsequently applied the fitted GAM to predict litterfall production under historical climate data (1970–2000 baseline) and future climate scenarios (SSP2‐4.5 and SSP5‐8.5) derived from CMIP6. Predictions were generated for four periods: 2021–2040, 2041–2060, 2061–2080, and 2081–2100. Changes in litterfall production due to climate were quantified relative to the historical climate baseline (1970–2000) to isolate climate‐driven effects on boreal litterfall production. Considering that different datasets have different resolutions, we uniformly resampled using the bilinear interpolation method to the same spatial resolution of 5 km to ensure data consistency. All spatial analyses were performed using the “raster” and “sf” packages (Pebesma 2018; Hijmans 2020).

Spatial autocorrelation is a common problem in spatial data analysis, and failure to account for it may result in an overestimation of the model's predictive performance (Ploton et al. 2020; Cai et al. 2023). To address this, we used Moran's *I* and semi‐variograms to identify spatial autocorrelation patterns in our litterfall production data. Our analysis revealed that spatial autocorrelation had a negligible effect on GAM results (Figure S4; Moran's *I* = 0.048, *p* = 0.260), suggesting that the selected model effectively captured the spatial structure in the litterfall data.

All statistical analyses and visualizations were performed using R statistical software (version 4.3.2; R Core Team 2023).

## 3 Results

### 3.1 Litterfall Production of Boreal Forests Across PFTs

**FIGURE 2:**
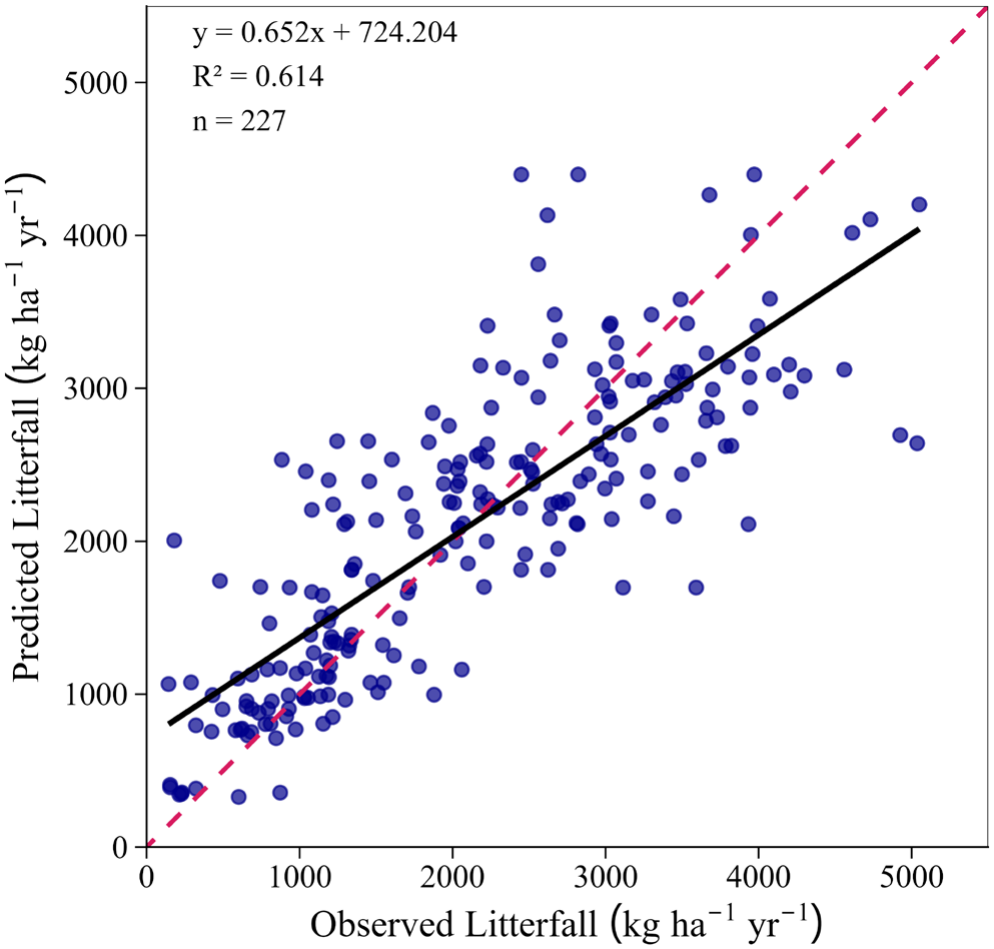
Predicted annual litterfall production plotted against observed litterfall production resulting from the selected generalized additive model. The dashed red line indicates 1:1 reference line and the solid black line represents the fitted regression line between predicted and observed values. *n* denotes the number of observations after removing the missing values for stand age.

The mean estimated litterfall production across boreal forests extracted from the literature published between 1974 and 2024 was 1959 kg ha^−1^ year^−1^ (±1206 kg ha^−1^ year^−1^). The selected GAM model explained 67% of the variation in litterfall production across the boreal forests. The model tended to overestimate litterfall production at very low or near zero observed litterfall values, while underestimating it at higher observed values (adjusted *R* ^2^ = 0.614; Figure 2). The model revealed that litterfall production was significantly influenced by PFTs, stand age, latitude, forest management types, and climatic factors (Table S5).

**FIGURE 3:**
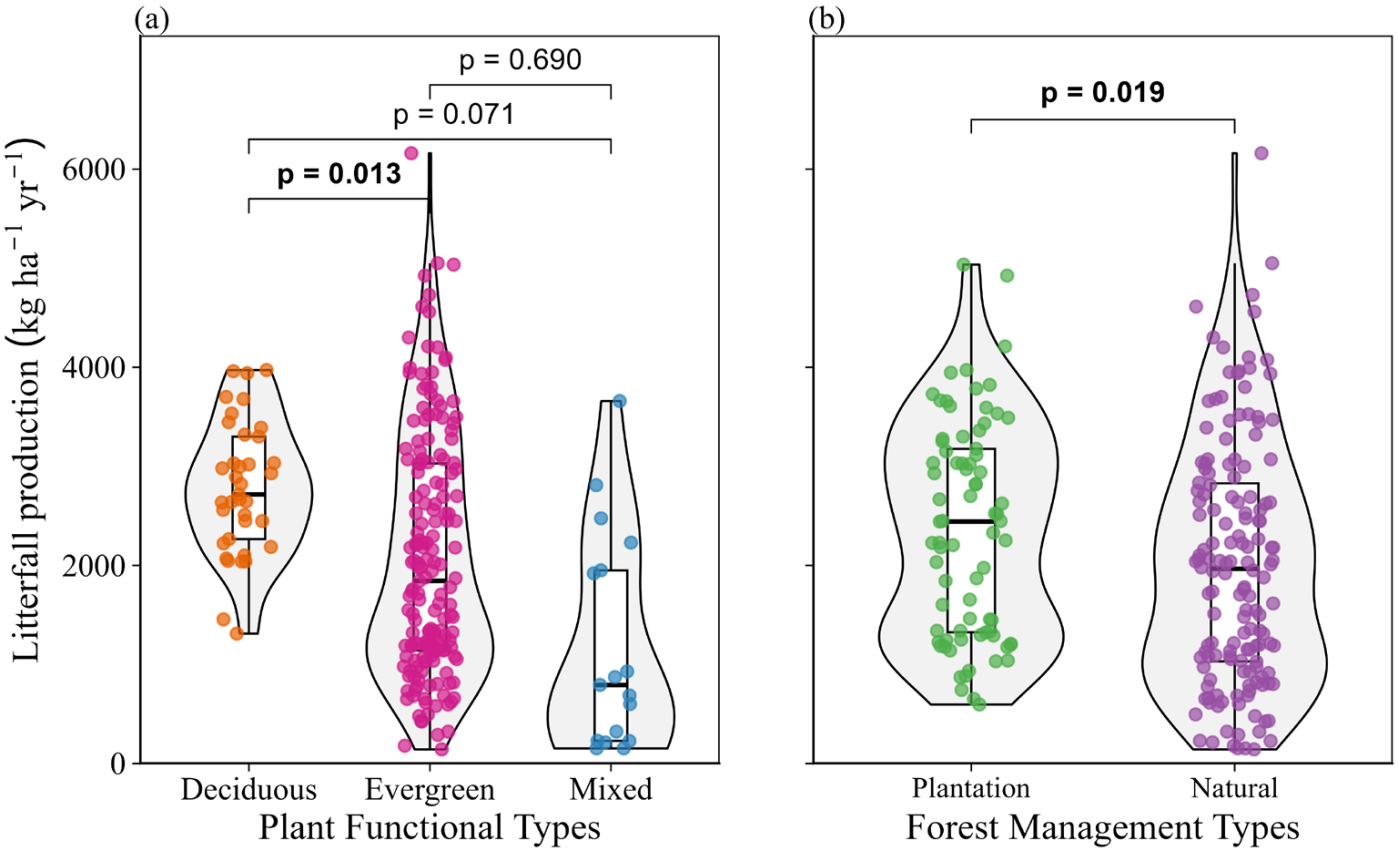
Annual litterfall production for different (a) plant functional types and (b) forest management types. Boxplots show the median and the 25% and 75% quantiles. Each point represents individual observations of litterfall production. *p* values show statistically significant differences between the groups.

PFTs had a significant and varied influence on litterfall (Figure 3a). Deciduous forests had greater litterfall production than that of evergreen forests (*p* < 0.05). Although litterfall production in deciduous forests was higher than in mixed forests, this difference was not statistically significant (*p* = 0.071). Litterfall production did not differ significantly between evergreen and mixed forests. In addition, for all forest ages, plantation‐managed forests had significantly greater litterfall production than natural forests (*p* < 0.05; Figure 3b).

**FIGURE 4:**
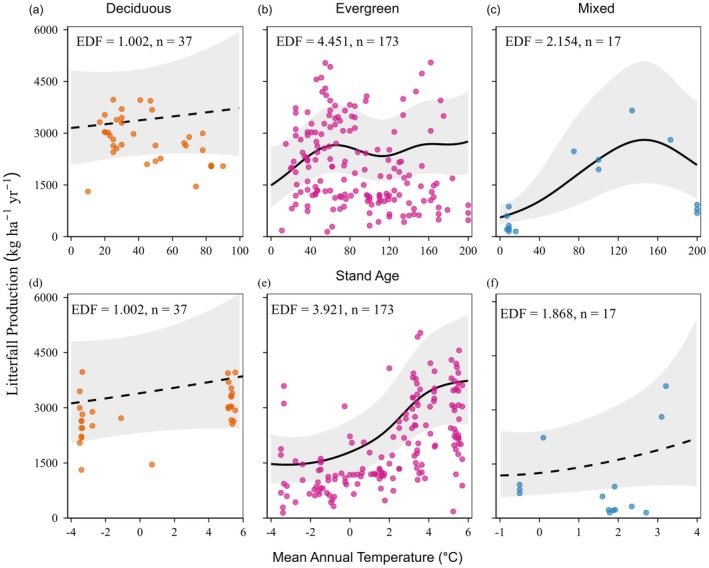
Generalized additive model (GAM) showing litterfall production relationships with (a–c) stand age and (d–f) mean annual temperature across plant functional types (orange = deciduous forests, pink = evergreen forests, blue = mixed forests). Solid lines indicate statistically significant relationships (*p* < 0.05), and dashed lines indicate nonsignificant relationships (*p* > 0.05). Shaded areas represent the 95% confidence intervals of the optimal GAM model. Effective degrees of freedom (EDF) indicate the complexity of the smoothing term; EDF = 1 indicates a linear relationship. Note that the sample sizes for deciduous (*n* = 37) and mixed (*n* = 17) forests are smaller than evergreen forests (*n* = 173) after accounting for missing stand age data.

The effects of stand age on litterfall production also differed among PFTs. Litterfall production in deciduous forests showed a linear pattern (effective degrees of freedom, EDF = 1.002), whereas evergreen (EDF = 4.451) and mixed forests (EDF = 2.154) displayed nonlinear responses (Figure 4a–c). As both evergreen and mixed forests' EDF values exceeded 1 but remained below the maximum EDF (*k* = 9), this suggested complex forest stand age‐related patterns without overfitting the model. In mixed forests, litterfall increased significantly with stand age up to approximately 150 years old, followed by a decline (*p* < 0.001; Figure 4c). Evergreen forests also showed a significant increase but only up to 60 years of age, after which they declined (*p* < 0.05; Figure 4b). However, in deciduous forests, there was no significant relationship between litterfall and stand age (*p* = 0.519; Figure 4a).

The response of litterfall to MAT was PFT‐specific. In deciduous forests, litterfall production exhibited a linear trend (EDF = 1.002; Figure 4d), while evergreen (EDF = 3.921) and mixed forests (EDF = 1.868) showed moderate to near‐nonlinear responses (Figure 4e,f). In evergreen forests, litterfall production showed a sigmoidal pattern with MAT (EDF = 3.921, *p* < 0.001). Litterfall production increased from −1°C to 5°C and plateaued near the lowest and highest temperatures (Figure 4e). In contrast, our model did not detect any significant effects of MAT on litterfall production in deciduous or mixed forests (Figure 4d–f).

**FIGURE 5:**
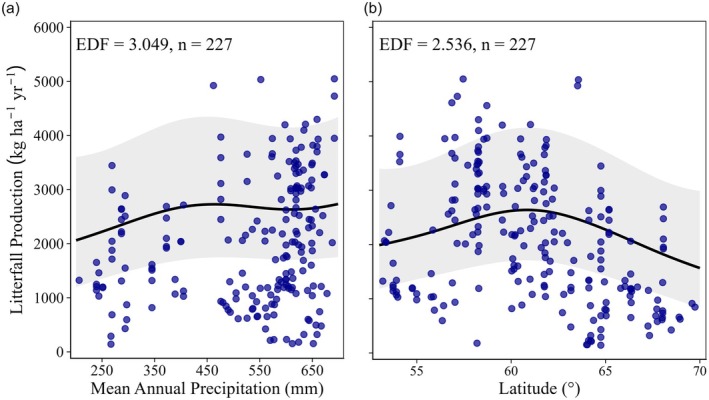
Generalized additive model (GAM) showing relationships between litterfall production and (a) mean annual precipitation and (b) latitude. Shaded areas represent the 95% confidence intervals of the selected GAM model. The effective degrees of freedom (EDF) indicate the complexity of the smoothing term; EDF = 1 indicates a linear relationship. *n* denotes the number of observations after removing the missing values for stand age.

Total annual litterfall production across all PFTs increased significantly with MAP, peaking near 500 mm annual precipitation before stabilizing or slightly declining (EDF = 3.049, *p* < 0.005; Figure 5a). In deciduous forests, litterfall production decreased with increasing MAP, but the effect was not significant (EDF = 1.002, *p* = 0.114; Figure S5a). In evergreen forests, litterfall production initially declined with MAP up to 500 mm precipitation, followed by an increase at higher precipitation (EDF = 4.134, *p* < 0.001; Figure S5b). Mixed forests exhibited a significant decrease in litterfall production with higher precipitation (EDF = 3.438, *p* < 0.001; Figure S5c). Litterfall production decreased significantly with latitude (EDF = 2.536, *p* < 0.05; Figure 5b). Forests located beyond 60°N exhibited the lower litterfall production (1790.442 kg ha^−1^ year^−1^) compared to forests located below 60°N (2566.650 kg ha^−1^ year^−1^).

### 3.2 Boreal Litterfall Production Patterns Under Historical (Baseline) and Future Climate Warming Scenarios

**FIGURE 6:**
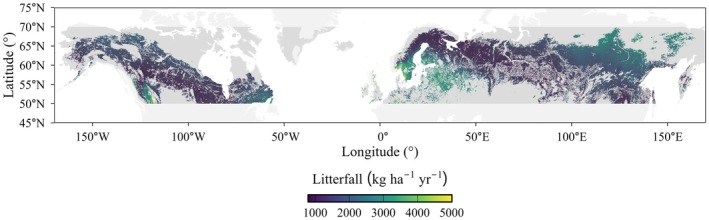
Spatial variability in boreal litterfall production (kg ha^−1^ year^−1^) predicted by GAM based on historical climates (mean annual temperature (MAT) and mean annual precipitation (MAP)) for the period of 1970–2000 from WorldClim version 2.1 data. The map was projected to 5 km spatial resolution.

Our analysis revealed distinct spatial variability in litterfall production across the boreal zone (Figure 6). Specifically, litterfall production was higher in parts of southern Fennoscandia, northern and eastern Canada, far‐eastern Siberia, and southern parts of European Russia, and was substantially lower in interior Alaska, north‐central continental regions of Canada, northern Europe, central Siberia, and central parts of European Russia.

**FIGURE 7:**
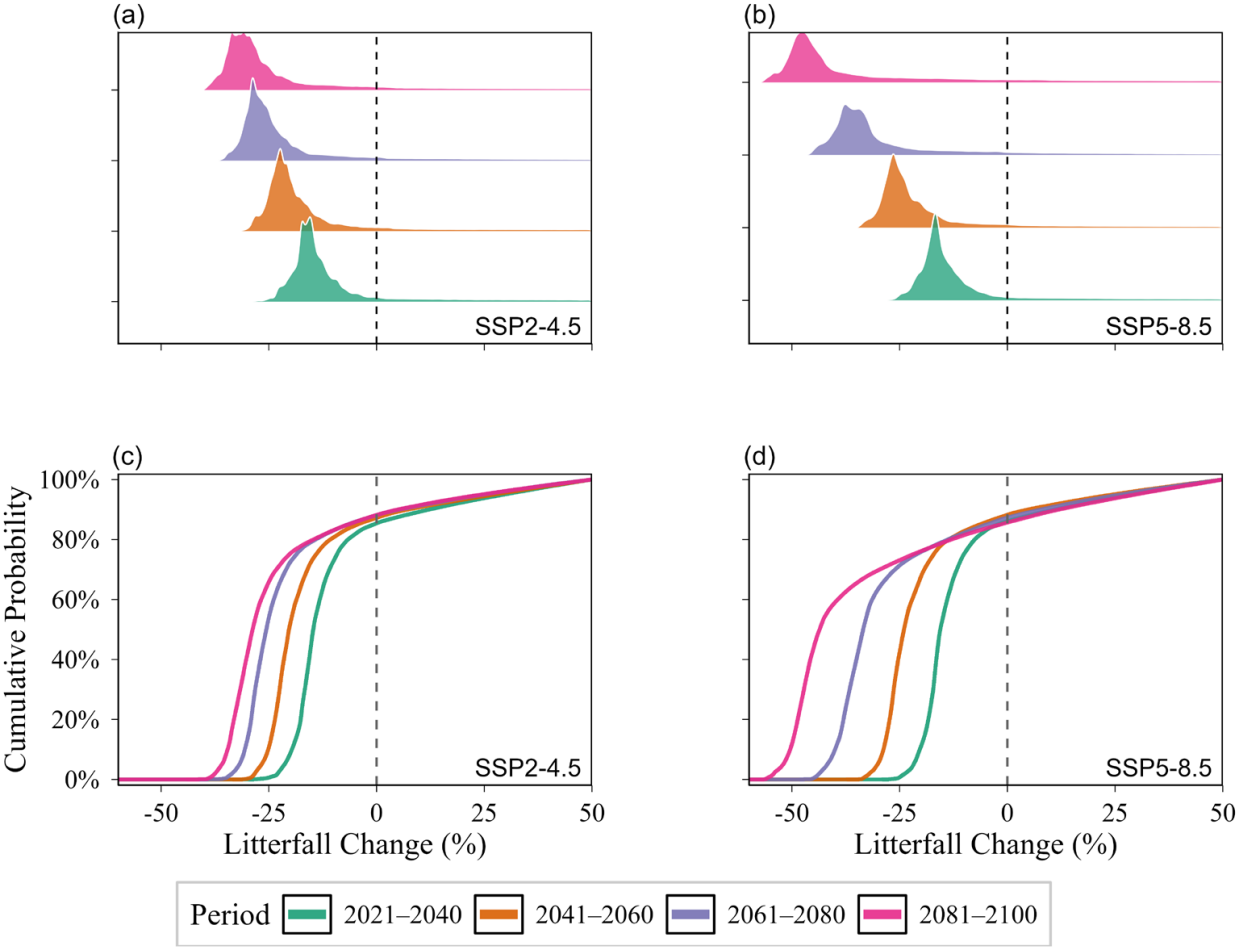
Probability density distribution (a, b) and cumulative distribution function (c, d) of projected relative litterfall production changes (%) under SSP2‐4.5 (left) and SSP5‐8.5 (right) across four time periods: 2021–2040, 2041–2060, 2061–2080, and 2081–2100.

Projected changes revealed consistent and pronounced temporal trends in litterfall production across 2021–2100 periods with probability density distributions showing a clear temporal trajectory of declining litterfall production in response to climate warming (Figure 7a,b). Declines in litterfall production were contingent upon emission scenarios and different time periods. Under both scenarios, litterfall production declined during each successive 20‐year period, with the magnitude of the reductions becoming progressively more pronounced over time. By the end of the 21st century (2081–2100), under the high‐emission SSP5‐8.5 scenario, the modal litterfall change dropped to below −25%, indicating a prevalent and significant decrease in litterfall production. This trend was further quantified by the cumulative distribution functions, which indicated that approximately 70% of the boreal forests are projected to experience litterfall declines exceeding −20% under SSP5‐8.5 by the end of the century, compared to the baseline (Figure 7c,d).

**FIGURE 8:**
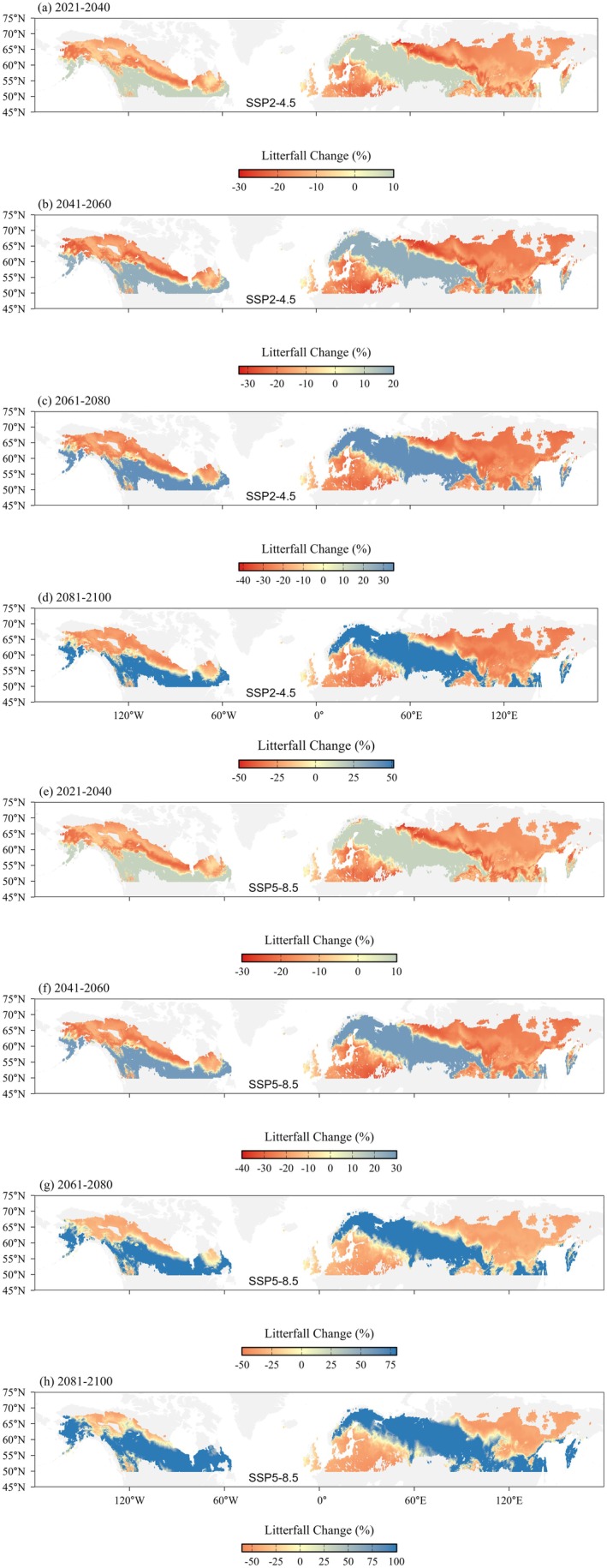
Spatiotemporal dynamics of projected relative changes in litterfall production (%) of boreal forest regions under two shared socioeconomic pathways (SSPs): (a–d) SSP2‐4.5 and (e–h) SSP5‐8.5. Results are shown for four time periods: 2021–2040, 2041–2060, 2061–2080, and 2081–2100. Shading indicates percentage change relative to a climate baseline (1970–2000), with blue colors representing increases in litterfall production and red colors indicating decreases.

Projected spatial patterns of changes in litterfall production revealed distinct spatial and temporal dynamics throughout the 21st century (Figure 8a–h). Both climate scenarios predicted widespread declines in litterfall production, particularly in central part of interior Alaska, northern‐central Canada, western and central Europe, and far‐eastern Siberia. These litterfall declines became more extensive and pronounced in later periods, especially under the SSP5‐8.5 scenario. Notably, the SSP5‐8.5 scenario resulted in greater decreases in litterfall with large areas experiencing decreases exceeding −50% compared to the SSP2‐4.5 (Figure 7a–d). In regions influenced by maritime climates along the Pacific coast of North America, northern part of Fennoscandian countries, and localized areas of Western Russia (Figure 8a–h), annual litterfall increased with increasing MAT, which contrasts with the boreal forests litterfall decline. The spatial patterns of decreases and increases in litterfall production are largely consistent across the climate scenarios. Nonetheless, the magnitude of projected changes is more pronounced in SSP5‐8.5 compared to SSP2‐4.5.

## 4 Discussion

This study provides a comprehensive synthesis and modeling of litterfall production across boreal forests, revealing how plant functional types (PFTs), stand age, forest management types, latitude, and climate collectively explain variation in litterfall production. By linking litterfall to climatic factors (MAT, MAP), we have provided insights into the sensitivity of litterfall production to future climate change. Our findings have important implications for understanding boreal forest C cycling and ecosystem stability under warming.

### 4.1 Litterfall Production Across PFTs and Forest Management Types

Consistent with our first hypothesis, litterfall production was significantly higher in deciduous forests than in evergreen forests. This pattern aligns with previous site‐level studies indicating that broadleaf tree species typically produce more litterfall than conifers (Chen et al. 2017), reflects the fundamental principles of leaf economic spectrum (Wright et al. 2004), and the fact that deciduous species lose all of their leaves in the autumn. Higher litterfall production in deciduous forests can be attributed to a greater variation in leaf traits, such as leaf longevity and tissue (Liu et al. 2020). Deciduous species exhibit an acquisitive strategy, and their shorter leaf lifespans and high specific leaf area allow for higher photosynthetic rates and a greater annual biomass turnover (Edwards et al. 2014). These deciduous plant traits drive a faster leaf turnover rate and ultimately result in greater litterfall production in deciduous forests (Reich et al. 2014). Evergreen species, in contrast, have a longer leaf lifespan and invest more resources into leaf protection and structural support, which results in lower photosynthetic and respiration rates.

Litterfall production in plantation managed forests was significantly higher than in natural forests. Plantation managed forests are often characterized by higher tree densities (Gao et al. 2023), which leads to more competition for light and resources. This, in turn, may promote faster leaf turnover as trees adjust their crown structure and allocate resources efficiently. In addition, leaves in plantation managed forests show a “faster investment‐return” resource utilization strategy, with high specific leaf area and low dry matter content, allowing them to capture resources efficiently and achieve faster turnover compared to the conservative strategy of natural forests (Zhang et al. 2024). Therefore, the combination of crown adjustments driven by management and resource‐acquisitive leaf traits likely explains these observed differences in litterfall production.

### 4.2 Stand Age Impacts Litterfall Production Across PFTs

While we did not directly use the global forest age dataset from Besnard et al. (2021), the stand ages in our dataset (7–250 years, mean of 85 years) align with the reported 0–300‐year range for boreal forest, indicating that boreal forests are mostly younger than 100 years. This is reflected in our dataset, where 64% of our sites are younger than 100 years, with the remaining sites representing mature stands. This age distribution supports a robust analysis of litterfall dynamics across stand age of boreal forests.

We found different age‐related patterns in litterfall production among PFTs. These findings partially confirmed our second hypothesis that litterfall production would peak during the intermediate stage before declining. In evergreen and mixed forests, the stand age‐related patterns of litterfall production followed single‐peak curves. This decline is likely linked to the natural aging process in plants, which reduces their growth efficiency and leaf turnover rates (Johnson and Abrams 2009). This pattern aligns well with stand age‐related trends in net primary productivity in boreal forests, which increases rapidly during early stand development before slowing as forests mature (Tang et al. 2014). In evergreen forests, litterfall production reached a maximum at about 60 years, which was similar to the age of the growth peak reported in previous studies (Besnard et al. 2018; Xu et al. 2020). In mixed forests, peak litterfall production occurred in older stand ages (around 150 years‐old), likely due to their high structural complexity (Yuan et al. 2018). The coexistence of species with contrasting structural, physiological, or phenological characteristics reduces the resource use competition among species and individual trees (Ackerly and Cornwell 2007). For instance, in mixed forests, evergreen tree species can access resources during the period when deciduous species have dropped their leaves, resulting in a longer annual period of resource availability (Kamiyama et al. 2014). In addition, coniferous species adapted to cold and harsh conditions can utilize available resources more efficiently, contributing to greater variation in tree size within the stand (Ali et al. 2020). Together, these mechanisms maintain forest productivity (Zohner and Renner 2019), thereby sustaining litterfall production at later stand ages. In contrast, deciduous forests did not exhibit any significant age‐related trend, but this may be due to insufficient sample sizes for young stands (< 20 years old).

The contrasting patterns between evergreen and deciduous forests are also supported by other studies on plant resource use efficiencies. In evergreen forests, resource use efficiency increases during early stand development and then declines with age, whereas deciduous forests tend to maintain more consistent resource use efficiency throughout stand aging (Xu et al. 2020). These resource use differences may also be attributed to differences in photosynthetic performance and stomatal conductance between the two forest types (Lusk et al. 2003; Klein and Ramon 2019).

Our findings highlight that litterfall production in boreal forests is strongly influenced by the interaction between stand age and PFTs. This underscores the importance of incorporating stand age into the predictive models, as neglecting this factor can introduce systematic bias in litterfall production estimates. While boreal evergreen and mixed forests followed a hump‐shaped pattern with stand age, deciduous forests remained relatively stable. This further emphasizes the critical need to consider species composition and ecological traits when modeling and predicting C and nutrient cycling across forest age gradients.

### 4.3 MAT Determines Litterfall Production Across PFTs

Our study revealed that different PFTs showed different responses to MAT for litterfall production, which partially supports our third hypothesis. In evergreen forests, litterfall production substantially increased with MAT up to a 5°C thermal threshold before leveling off. This threshold aligns with physiological evidence that tree productivity in evergreen conifer forests is constrained at low temperatures. For example, gross primary productivity in evergreen conifer forest is gradually inhibited at temperatures below approximately 6°C (±2.6°C) (Stettz et al. 2022). Similarly, in the southern margin of a cold temperate coniferous forest, radial growth is sensitive to daily mean temperatures below 4°C–5.5°C, reflecting the minimum requirements for sap flow, photosynthesis, and tree germination (Li et al. 2021).

In high‐latitude boreal forests, increasing temperatures directly influence tree growth through effects on photosynthesis and heterotrophic respiration rates (D'Orangeville et al. 2018). Moderate temperature increases may stimulate vegetation growth by increasing the length of the plant growing season and enhancing photosynthetic activity (Zhang et al. 2022). However, in regions prone to droughts, rising temperatures might reduce productivity when the tree closes its stomata to conserve water and may also increase mortality rates (Liu et al. 2023). These mechanisms likely explain why evergreen forests showed temperature‐driven increases in litterfall production up to a threshold, beyond which physiological constraints and disturbance risks may limit future productivity.

Deciduous and mixed forests showed nonsignificant litterfall responses to MAT in the global model. Failure to detect significant trends in litterfall production with MAT in this analysis does not imply that none exist. The separate climate‐focused GAM models for deciduous, mixed, and evergreen forests that we conducted to address this revealed significant nonlinear responses to MAT in deciduous and mixed forests (Figure S6a–c). These results suggest that the effect was masked in the global model, likely due to differential responses across PFTs or the limited data coverage across the temperature gradient. In contrast, evergreen forests showed consistent temperature responses in both climate‐focused and global models (Figure S6b), indicating greater robustness in their climate sensitivity. These PFT‐specific litterfall production responses highlight the importance of incorporating physiological thresholds, phenology, and environmental stressors into models projecting boreal forest C cycling under a warming climate.

### 4.4 Projected Widespread Declines in Litterfall Production Under Future Climate Warming Scenarios

Projected changes in litterfall production were assessed against our hypothesis that litterfall would increase under SSP2‐4.5 due to extended growing seasons, but decline under SSP5‐8.5 as warming surpasses the thermal tolerance of boreal tree species. Our results partially support this hypothesis. While increases in litterfall were observed in colder regions, such as northern Scandinavia, Pacific coastal forests of North America, and western Interior Alaska, widespread declines dominated much of the boreal biome, particularly under SSP5‐8.5, where reductions exceeded 50% in interior continental regions. In contrast, regions with historically high litterfall, including western and central Europe, northeastern Canada, and far eastern Russia, exhibited consistent declines. These patterns align with previous projections of boreal biomass dynamics (Xu and Hisano 2024).

These contrasting responses reflect a shift from temperature limitation to physiological stress. In colder and wetter regions, moderate warming can alleviate thermal constraints and enhance productivity, leading to increased biomass and litterfall (D'Orangeville et al. 2016; Schaphoff et al. 2016; Aakala et al. 2018; Sulla‐Menashe et al. 2018). However, under more intense warming, elevated temperatures and vapor pressure deficit (VPD) intensify plant water stress and suppress photosynthesis, as both photosynthetic rates and stomatal conductance consistently decline with increasing VPD (Reich et al. 2018; Yuan et al. 2019; Grossiord et al. 2020; Eze et al. 2024). As an organ directly exposed to environmental conditions, leaves are particularly sensitive to light and temperature, especially because photosystem II (PSII), a heat‐sensitive component of the photosynthetic apparatus, is highly vulnerable to heat and high irradiation stress during C assimilation (Dongsansuk et al. 2013; Jiang et al. 2021). Under these conditions, photoinhibition becomes a dominant constraint, as PSII loses efficiency when temperatures exceed critical thresholds (Weis and Berry 1987; Geange et al. 2021). The resulting reduction in C gain relative to C loss suppresses growth and accelerates leaf senescence through chlorophyll degradation and nutrient resorption, promoting earlier leaf abscission and ultimately reducing litterfall.

Lower litterfall production may limit soil organic C accumulation, while higher temperatures accelerate soil respiration (Carey et al. 2016). Together, these processes may weaken the boreal C sink and contribute to positive climate feedbacks. Given that boreal ecosystems store nearly one‐third of global terrestrial C (Pan et al. 2011; Scharlemann et al. 2014; Bradshaw and Warkentin 2015), continued declines in litterfall under high emission scenarios could have substantial implications. Evidence indicates that some regions, including western Canada and Siberia, may already be shifting from C sinks to sources (Dolman et al. 2012; Gauthier et al. 2015; Tompalski et al. 2024).

Overall, boreal forest responses to warming are nonlinear and threshold‐dependent. Moderate warming can enhance productivity in cold‐limited regions, but higher temperatures trigger photoinhibition and water stress that suppress C assimilation and litterfall. These shifts threaten the strength of the boreal C sink under high‐emission trajectories, underscoring its functioning and its critical role in the global C cycle.

### 4.5 Further Research Considerations

Our future projection models were designed to isolate the direct effects of climate factors on litterfall production under a static vegetation scenario. By holding PFTs, forest management types, and stand age constant, we provided a crucial baseline for direct climate forcing. However, we recognize that the boreal forest is a dynamic system where climate change will also drive shifts in vegetation composition, intensify disturbance regimes, and alter successional trajectories that could not be completely addressed with our models here. Additionally, our model could not separately project the future for the different PFTs. We clearly demonstrated PFT‐specific responses to climatic drivers and have suggested that future projections of litterfall production would differ for the PFTs. Therefore, we urge the development of a new generation of models that integrate dynamic global vegetation models with empirical litterfall production data. Such models could simulate the coupled effects of climate change, shifts in vegetation cover from evergreen to deciduous, and disturbances on future C cycling, providing a more holistic and realistic projection.

## 5 Conclusion

Our study demonstrates the critical role of incorporating Plant Functional Types and their physiological thresholds when modeling forest litterfall dynamics. While our results focus primarily on the boreal forests, the broader applicability of these findings lies in the fact that PFT‐specific differences in leaf longevity, C allocation, and nutrient cycling represent fundamental plant strategies that are conserved across forest biomes. In addition, the observed relationships between litterfall production and stand age and temperature reflect general forest developmental processes and physiological constraints that regulate productivity globally, although the magnitude and threshold values may vary among regions. By synthesizing biome‐scale patterns, we show that litterfall production is not a simple function of climate but is governed by the intricate interplay of various attributes including climatic factors, stand age, and PFT‐specific physiological responses. Under future climate scenarios, our projections anticipate substantial declines in litterfall production across extensive areas of boreal forests. These suggest potential negative feedback on boreal forest productivity, which would alter soil organic matter, C stocks and decomposition rates, nutrient availability, and overall C sequestration capacity. Our findings highlight the importance of accounting for PFTs and physiological thresholds when investigating the litterfall dynamics in forests and the potential impact of climate warming on the contribution of litterfall to carbon and nutrient cycling.

## Author Contributions

**Wai Phyo Thu:** conceptualization (equal), data curation (equal), formal analysis (equal), software (equal), visualization (equal), writing – original draft (equal). **Mark Jun M. Alcantara:** formal analysis (equal), software (equal), visualization (equal), writing – review and editing (equal). **Gbadamassi G. O. Dossa:** conceptualization (equal), data curation (equal), funding acquisition (equal), methodology (equal), validation (equal), writing – review and editing (equal). **Jill Thompson:** writing – review and editing (equal). **Douglas Schaefer:** conceptualization (equal), writing – review and editing (equal).

## Funding

This work was supported by National Natural Science Foundation of China (NSFC) (W2432022), Yunnan Province Government for Talents Program (E1YN101B01), Zhi Hui Yunnan Program (202203AM140024).

## Conflicts of Interest

The authors declare no conflicts of interest.

## Supporting information


**Dataset: S1** with a full list of references used for data compilation and plant functional type classification.

**Figure S1:** Number of data points by sampling year from 1969 to 2024. The dashed line marks the year 2000 for the reference. In total, 46.6% of data points were collected before 2000 and 53.4% after 2000.
**Figure S2:** Model diagnostics plots for the selected generalized additive model (GAM) fitted to litterfall production data (Model 6). The figure includes four panels: (i) quantile–quantile (QQ) plot of deviance residuals (upper left); (ii) deviance residuals against linear predictor values (upper right); (iii) histogram of deviance residuals (lower left); (iv) observed versus fitted values (lower right). Note that diagnostics were performed for all models and we did not find significant problems except for the top‐ranked model, which was underdispersed.
**Figure S3:** Generalized additive model (GAM) smoothing curves fitted to the partial effects of (a) mean annual temperature (MAT) and (b) mean annual precipitation (MAP) on litterfall production. Shaded areas indicate the 95% confidence intervals. Tick marks along the x‐axis (rug plot) indicate the sample sizes distribution across the climate gradient. TPRS denotes thin plate regression spline. Note that the results are derived from the climate‐focused model without separating plant functional types.
**Figure S4:** Spatial variogram of model residuals for litterfall production across the boreal biome. The empirical semivariance (blue points) shows the spatial autocorrelation structure at different distances. The red line shows a LOESS smooth trend illustrating the pattern of spatial autocorrelation. Gray dashed lines indicate the 95% confidence envelope calculated from the semivariance values and number of point pairs. The relatively flat profile of semivariance across distances suggests that spatial autocorrelation has negligible influence on our model results.
**Figure S5:** Generalized additive model (GAM) smoothing curves fitted to the partial effects of mean annual precipitation (MAP) on litterfall production in (a) deciduous forest, (b) evergreen forest, and (c) mixed forest. Tick marks along the x‐axis (rug plot) indicate the sample sizes distribution across the precipitation gradient. TPRS denotes thin plate regression spline. Note that the results are derived from the MAP‐focused climate model.
**Figure S6:** Generalized additive model (GAM) smoothing curves fitted to the partial effects of mean annual temperature (MAT) on litterfall production in (a) deciduous forest and (b) evergreen forest. Tick marks along the x‐axis (rug plot) indicate the sample sizes distribution across the stand age. TPRS denotes thin plate regression spline. Note that the results are derived from the MAT‐focused climate model.
**Table S1:** Classification of MODIS land cover types and corresponding ecosystems.
**Table S2:** Variance Inflation Factor (VIF) values for all variables included in the model. MAT represents mean annual temperature, MAP represents mean annual precipitation.
**Table S3:** Model selection results for the top seven generalized additive models (GAM) predicting litterfall production. Models are ranked by Akaike's Information Criterion (AIC), with DeltaAICc representing the difference from the top model. The selected best‐supported model (No. 6) is highlighted in bold.
**Table S4:** Generalized additive model (GAM) basic dimension (*k*) diagnostics for litterfall production.
**Table S5:** Summary of the selected GAM model for litterfall production.

## Data Availability

The data and code that support the findings of this study are openly available at https://doi.org/10.5281/zenodo.17841521.
