# Supplementary material for: Climate Warming Will Reduce Boreal Forest Litterfall, but the Response Differs Among Plant Functional Types
Source: Ecol Evol. 2026 May 27;16(6):e73726. doi: 10.1002/ece3.73726 (PMC13239982; doi:10.1002/ece3.73726)
Supplement: Supplementary file 2 — Figure S1: Number of data points by sampling year from 1969 to 2024. The dashed line marks the year 2000 for the reference. In total, 46.6% of data points were collected before 2000 and 53.4% after 2000. Figure S2: Model diagnostics plots for the selected generalized additive model (GAM) fitted to litterfall production data (Model 6). The figure includes four panels: (i) quantile–quantile (QQ) plot of deviance residuals (upper left); (ii) deviance residuals against linear predictor values (upper right); (iii) histogram of deviance residuals (lower left); (iv) observed versus fitted values (lower right). Note that diagnostics were performed for all models and we did not find significant problems except for the top‐ranked model, which was underdispersed. Figure S3: Generalized additive model (GAM) smoothing curves fitted to the partial effects of (a) mean annual temperature (MAT) and (b) mean annual precipitation (MAP) on litterfall production. Shaded areas indicate the 95% confidence intervals. Tick marks along the x‐axis (rug plot) indicate the sample sizes distribution across the climate gradient. TPRS denotes thin plate regression spline. Note that the results are derived from the climate‐focused model without separating plant functional types. Figure S4: Spatial variogram of model residuals for litterfall production across the boreal biome. The empirical semivariance (blue points) shows the spatial autocorrelation structure at different distances. The red line shows a LOESS smooth trend illustrating the pattern of spatial autocorrelation. Gray dashed lines indicate the 95% confidence envelope calculated from the semivariance values and number of point pairs. The relatively flat profile of semivariance across distances suggests that spatial autocorrelation has negligible influence on our model results. Figure S5: Generalized additive model (GAM) smoothing curves fitted to the partial effects of mean annual precipitation (MAP) on litterfall production in (a) decidu [file ECE3-16-e73726-s001.docx]

Supplementary Information for

**Title: Climate warming will reduce boreal forest litterfall, but the response differs among plant functional types.**

Wai Phyo Thu ^a, b, c^, Mark Jun M. Alcantara ^a, b^, Gbadamassi G.O. Dossa ^a, b, *^, Jill Thompson ^d^, Douglas Schaefer ^e *^

^a^ Laboratory of Tropical Forest Ecology, Xishuangbanna Tropical Botanical Garden, Chinese Academy of Sciences

^b^ Yunnan Key Laboratory of Forest Ecosystem Stability and Global Change Response, Xishuangbanna Tropical Botanical Garden, Chinese Academy of Sciences, Mengla, Yunnan 666303, China

^c^ University of Chinese Academy of Sciences, Beijing 100049, China

^d^ UK Centre for Ecology & Hydrology Bush Estate, Penicuik, Midlothian, EH260QB, UK

^e^ Centre for Mountain Futures, Kunming Institute of Botany, Chinese Academy of Sciences, Kunming 650201, Yunnan, China

***Corresponding authors:**

Gbadamassi G.O. Dossa, email: [dgbadamassi@gmail.com](mailto:dgbadamassi@gmail.com)

Douglas Schaefer, email: [schaefer@mail.kib.ac.cn](mailto:schaefer@mail.kib.ac.cn)

**This file includes:**

Figures S1 to S6; Tables S1 to S4

Other supporting materials for this manuscript include the following:

Dataset S1 with a full list of references used for data compilation and plant functional type classification

**
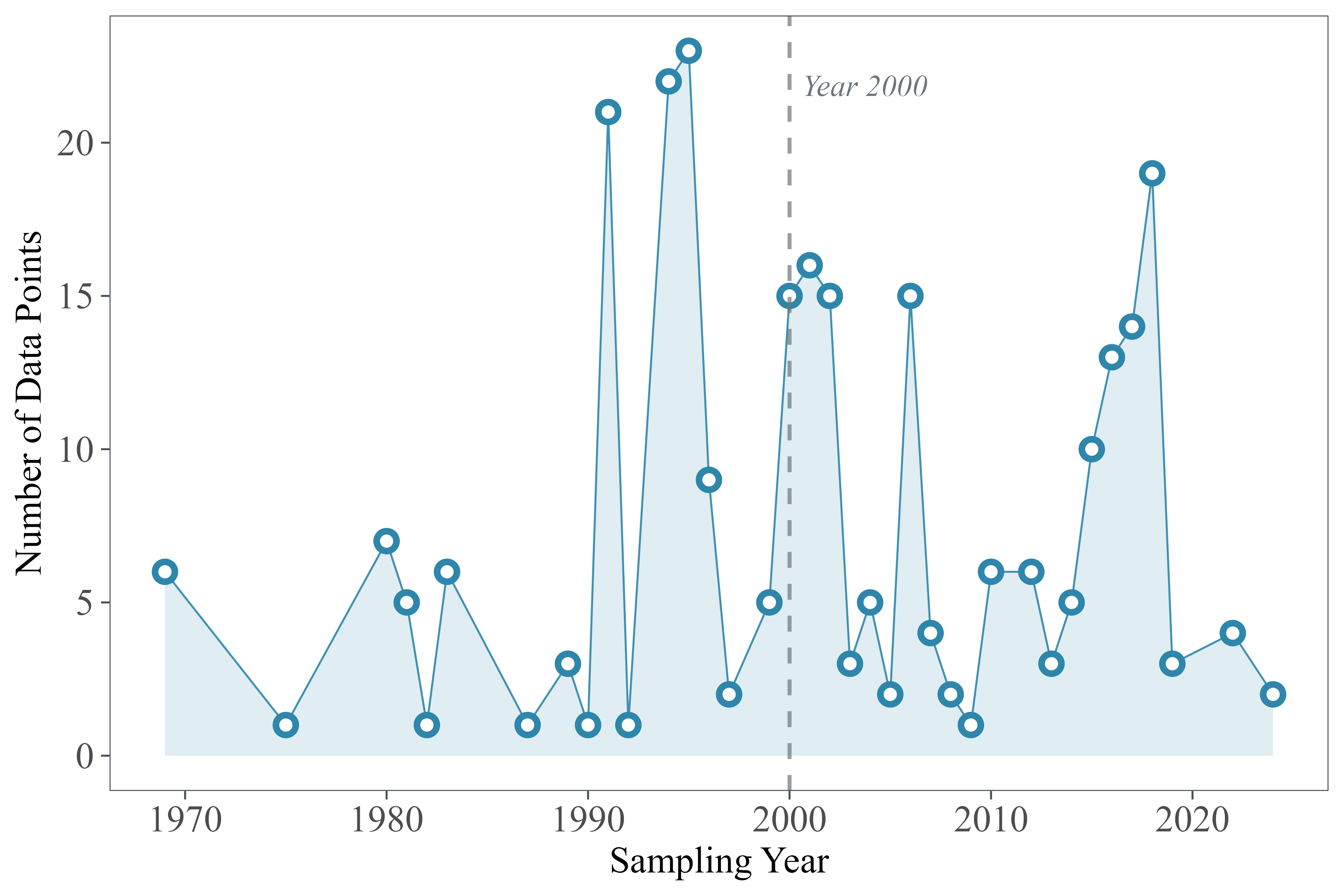
**

FIGURE S1. Number of data points by sampling year from 1969 to 2024. The dashed line marks the year 2000 for the reference. In total, 46.6% of data points were collected before 2000 and 53.4% after 2000.

**
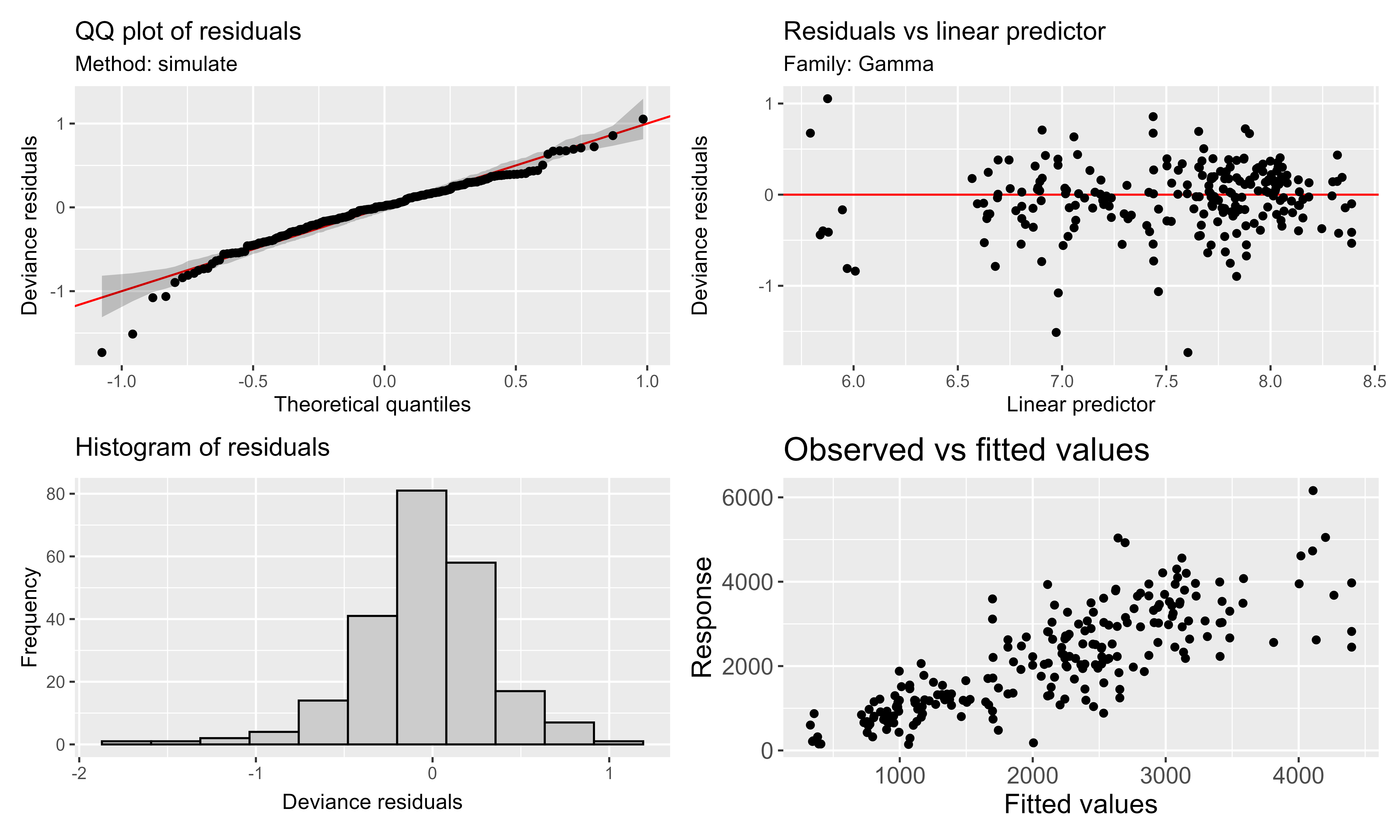
**

FIGURE S2. Model diagnostics plots for the selected generalized additive model (GAM) fitted to litterfall production data (Model 6). The figure includes four panels: (i) quantile-quantile (QQ) plot of deviance residuals (upper left); (ii) deviance residuals against linear predictor values (upper right); (iii) histogram of deviance residuals (lower left); (iv) observed versus fitted values (lower right). Note that diagnostics were performed for all models and we did not find significant problems except for the top-ranked model, which was underdispersed.


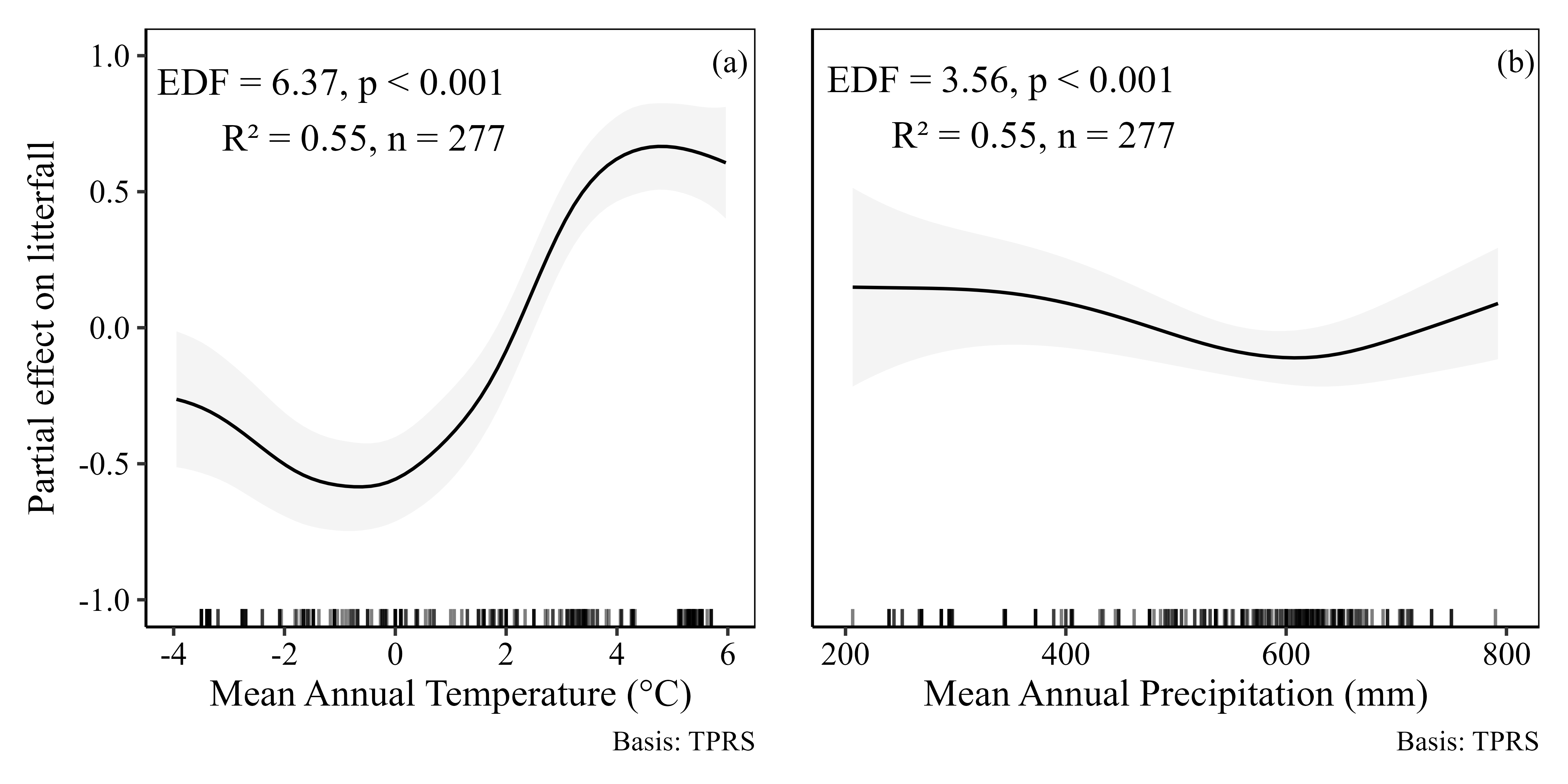


FIGURE S3. Generalized additive model (GAM) smoothing curves fitted to the partial effects of (a) mean annual temperature (MAT) and (b) mean annual precipitation (MAP) on litterfall production. Shaded areas indicate the 95% confidence intervals. Tick marks along the x-axis (rug plot) indicate the sample sizes distribution across the climate gradient. TPRS denotes thin plate regression spline. Note that the results are derived from the climate-focused model without separating plant functional types.


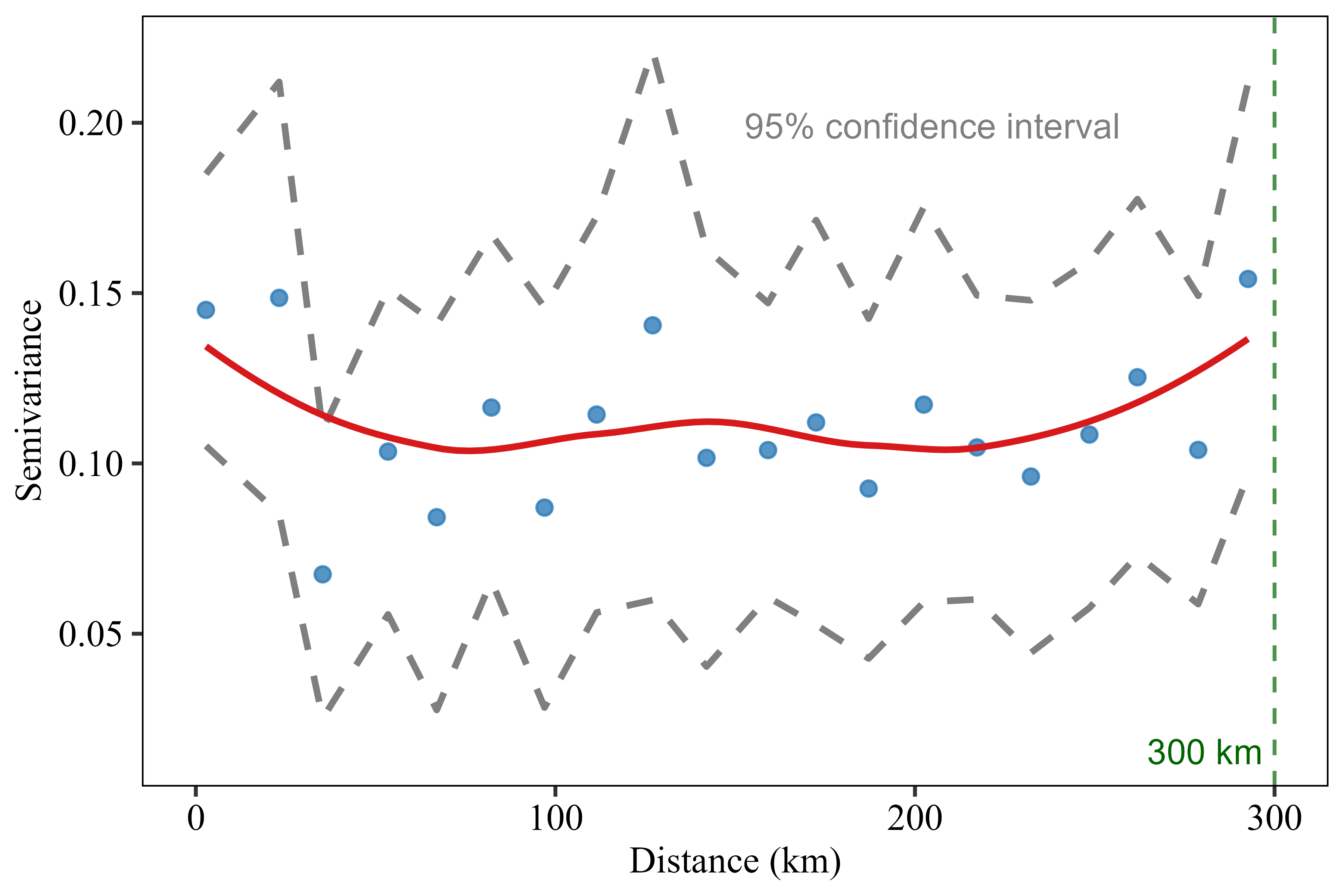


FIGURE S4. Spatial variogram of model residuals for litterfall production across the boreal biome. The empirical semivariance (blue points) shows the spatial autocorrelation structure at different distances. The red line shows a LOESS smooth trend illustrating the pattern of spatial autocorrelation. Gray dashed lines indicate the 95% confidence envelope calculated from the semivariance values and number of point pairs. The relatively flat profile of semivariance across distances suggests that spatial autocorrelation has negligible influence on our model results.


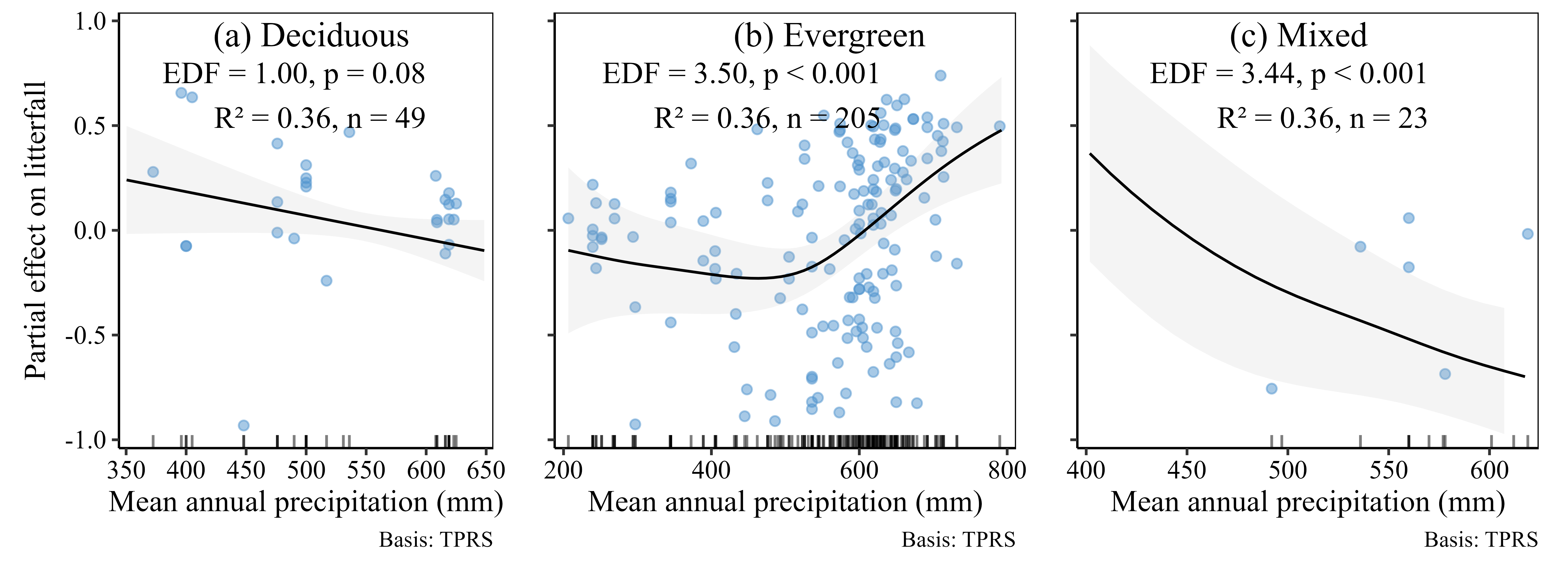


FIGURE S5. Generalized additive model (GAM) smoothing curves fitted to the partial effects of mean annual precipitation (MAP) on litterfall production in (a) deciduous forest, (b) evergreen forest, and (c) mixed forest. Tick marks along the x-axis (rug plot) indicate the sample sizes distribution across the precipitation gradient. TPRS denotes thin plate regression spline. Note that the results are derived from the MAP-focused climate model.


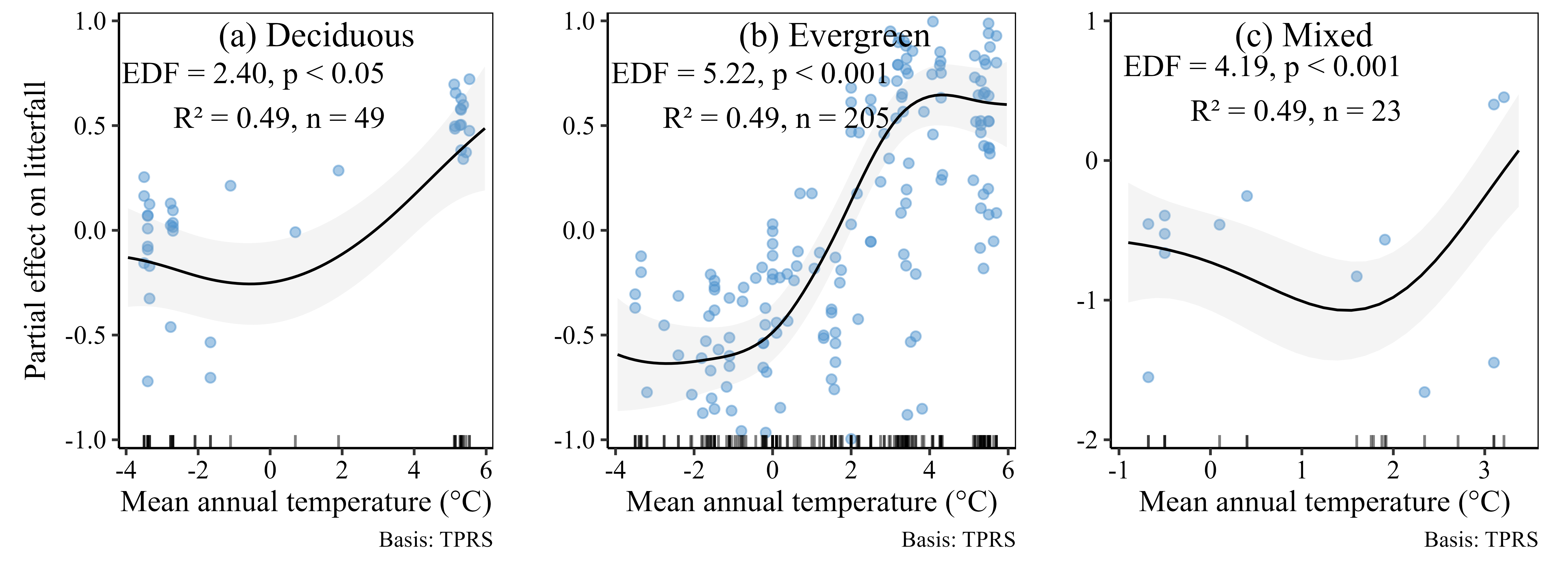


FIGURE S6. Generalized additive model (GAM) smoothing curves fitted to the partial effects of mean annual temperature (MAT) on litterfall production in (a) deciduous forest and (b) evergreen forest. Tick marks along the x-axis (rug plot) indicate the sample sizes distribution across the stand age. TPRS denotes thin plate regression spline. Note that the results are derived from the MAT-focused climate model.

TABLE S1. Classification of MODIS land cover types and corresponding ecosystems

| **Value** | **Land cover classification** | **Forest classification in this study** |
| --- | --- | --- |
| 0 | Water bodies | Non-forest |
| 1 | Evergreen Needleleaf Forests | Forests |
| 2 | Evergreen Broadleaf Forests | Forests |
| 3 | Deciduous Needleleaf Forests | Forests |
| 4 | Deciduous Broadleaf Forests | Forests |
| 5 | Shrub | Non-forest |
| 6 | Grass | Non-forest |
| 7 | Cereal croplands | Non-forest |
| 8 | Broadleaf croplands | Non-forest |
| 9 | Urban and built-up lands | Non-forest |
| 10 | Non-vegetated lands | Non-forest |
| 11 | Permanent snow and ice | Non-forest |

TABLE S2. Variance Inflation Factor (VIF) values for all variables included in the model.

MAT represents mean annual temperature, MAP represents mean annual precipitation.

| **Predictor** | **Generalized VIF** | **Degrees of Freedom** | **Adjusted GVIF** |
| --- | --- | --- | --- |
| Plant functional types | 1.54 | 2 | 1.11 |
| Forest management types | 1.24 | 1 | 1.11 |
| MAT | 2.73 | 1 | 1.65 |
| MAP | 1.84 | 1 | 1.36 |
| Stand age | 1.52 | 1 | 1.23 |
| Latitude | 1.37 | 1 | 1.17 |
| Elevation | 1.58 | 1 | 1.26 |

TABLE S3. Model selection results for the top seven generalized additive models (GAM) predicting litterfall production. Models are ranked by Akaike’s Information Criterion (AIC), with DeltaAICc representing the difference from the top model. The selected best-supported model (No.6) is highlighted in bold.

| **No.** | **Model Parameters** | **AICc** | **DeltaAICc** | **R^2^** | **DevExplained** | **n** |
| --- | --- | --- | --- | --- | --- | --- |
| 1 | PFT + s(Age, by = PFT) + s(MAP) + s(MAT, by = PFT) + s(Country) | 3670.773 | 0.000 | 0.622 | 0.657 | 227 |
| 2 | FM + PFT + s(MAP ) + s(MAT, by = PFT) + s(Country) | 3671.500 | 0.727 | 0.585 | 0.643 | 227 |
| 3 | PFT + s(MAP ) + s(MAT, by = PFT) + s(Country) | 3671.610 | 0.837 | 0.580 | 0.627 | 227 |
| 4 | FM + PFT + s(MAP) + s(MAT, by = PFT) + s(Country) | 3671.800 | 1.027 | 0.580 | 0.628 | 227 |
| 5 | PFT+ s(MAT, by = PFT) + s(Country) | 3671.938 | 1.165 | 0.569 | 0.617 | 227 |
| 6 | **FM + PFT + s(Age, by = PFT) + s(Latitude) + s(MAP) + s(MAT, by = PFT) + s(Country)** | **3672.250** | **1.477** | **0.614** | **0.667** | **227** |
| 7 | PFT + s(Latitude) + s(MAP) + s(MAT, by = PFT) + s(Country) | 3672.258 | 1.485 | 0.582 | 0.637 | 227 |

Notes:

- Model terms are abbreviated as follows: FM = forest management types; PFT = plant functional types; MAT = mean annual temperature; MAP = mean annual precipitation; s() = thin-plate regression splines.
- Country was included as a random intercept.
- All models are GAMs fitted with a Gamma family and log link function.
- Model performance metrics include adjusted R-squared (R²); percentage of deviance explained (DevExplained), and the sample size (n = 227).
- Although Model 1 had the lowest AICc score, model 6 was selected as the best-supported model for inference due to its superior explanatory power, and inclusion of key predictive variables.

TABLE S4. Generalized additive model (GAM) basic dimension (k) diagnostics for litterfall production

| Smooth terms | k' | EDF | k-index | p-value |
| --- | --- | --- | --- | --- |
| s(Age):Functional type (Deciduous) | 9 | 1.002 | 1.009 | 0.628 |
| s(Age):Functional type (Evergreen) | 9 | 4.451 | 1.009 | 0.603 |
| s(Age):Functional type (Mixed) | 9 | 2.154 | 1.009 | 0.658 |
| s(Latitude) | 9 | 2.536 | 1.015 | 0.688 |
| s(MAP) | 9 | 3.049 | 0.915 | 0.145 |
| s(MAT):Functional type (Deciduous) | 9 | 1.002 | 0.874 | 0.030 |
| s(MAT):Functional type (Evergreen) | 9 | 3.921 | 0.874 | 0.043 |
| s(MAT):Functional type (Mixed) | 9 | 1.868 | 0.874 | 0.038 |

Notes:

- The table shows the maximum possible degrees of freedom for each smooth term (k'), the estimated degrees of freedom (EDF), the k-index, and its associated p-value for each smooth term. A k-index value below 1 suggests the term may be undersmoothed, with a significant p-value (p < 0.05) providing statistical evidence. The apparent undersmoothing observed for the s(MAT) smooths by plant function types is likely due to uneven sample sizes across the temperature gradient, whereas the available degrees of freedom were sufficient for the other model terms.

TABLE S5. Summary of the selected GAM model for litterfall production

| Parametric coefficient | Level | Estimate | Std. Error | t value | p-value |
| --- | --- | --- | --- | --- | --- |
| (Intercept) | (Plantation, Deciduous) | 8.126 | 0.169 | 47.947 | 0.000^***^ |
| Forest management types | Natural | -0.161 | 0.068 | -2.372 | 0.019* |
| Functional type | Evergreen | -0.457 | 0.136 | -3.366 | 0.001^**^ |
| Functional type | Mixed | -0.749 | 0.304 | -2.459 | 0.015^*^ |

| Smooth terms | EDF | Ref.df | F | p-value |
| --- | --- | --- | --- | --- |
| s(Age):Functional type (Deciduous) | 1.002 | 1.003 | 0.415 | 0.519 |
| s(Age):Functional type (Evergreen) | 4.451 | 5.471 | 2.519 | 0.039^*^ |
| s(Age):Functional type (Mixed) | 2.154 | 2.464 | 11.370 | 0.000^***^ |
| s(Latitude) | 2.536 | 3.094 | 3.064 | 0.029^*^ |
| s(MAP) | 3.049 | 3.795 | 3.694 | 0.005^**^ |
| s(MAT):Functional type (Deciduous) | 1.002 | 1.003 | 0.809 | 0.369 |
| s(MAT):Functional type (Evergreen) | 3.921 | 4.866 | 6.566 | 0.000^***^ |
| s(MAT):Functional type (Mixed) | 1.866 | 2.046 | 0.678 | 0.364 |
| s(Country) | 5.335 | 8.000 | 3.607 | 0.000^***^ |

Model statistics: R-sq.(adj) = 0.614, Deviance explained = 66.7%, -REML = 1837.8, Scale est. = 0.13685, n = 227

**Notes:**

1. Parametric coefficients show the linear effects of categorical predictors on litterfall production. The intercept corresponds to Plantation managed forests and Deciduous forests. Positive estimates indicate an increase in litterfall relative to the reference, while negative estimates indicate a decrease.
2. Smooth terms show non-linear effects of continuous predictors on litterfall production. Effective degrees of freedom (EDF) quantify the flexibility of the smooth term:

- EDF =1 indicates a linear relationship
- EDF > 1 indicates a non-linear relationship, with higher values corresponding to greater complexity and stronger non-linearity.

1. p-value indicates the level of significance: *** p < 0.001, ** p < 0.01, * p < 0.05.
2. Country was included as a random effect to account for potential non-independence among observations within countries.
